# Joint ABS-UKCGG-CanGene-CanVar consensus regarding the use of CanRisk in clinical practice

**DOI:** 10.1038/s41416-024-02733-4

**Published:** 2024-06-04

**Authors:** Olga Tsoulaki, Marc Tischkowitz, Antonis C. Antoniou, Hannah Musgrave, Gillian Rea, Ashu Gandhi, Karina Cox, Tracey Irvine, Sue Holcombe, Diana Eccles, Clare Turnbull, Ramsey Cutress, Meeting Attendees, Avgi Andreou, Abdul Badran, Marion Bartlett, Cheryl Berlin, Kathie Binysh, Paul Brennan, Ruth Cleaver, Gemma Corbett, Rosemarie Davidson, Bianca DeSouza, Rachel Evers, Lorenzo Ficorella, Suzannah Fitzgerald, Andrea Forman, Fiona Gilbert, Rochelle Gold, Steph Greville-Heygate, Sarah Hamilton, Carrie Hammond, Rachel Hart, Lara Hawkes, Jude Hayward, Debbie Holliday, Antony Howell, Gillian Hutchison, Jacqui Jenkins, Rosalyn Jewell, Grace Kavanaugh, Zoe Kemp, Victoria Kiesel, Ajith Kumar, Fiona Lalloo, Zosia Miedzybrodzka, Jennie Murray, Sian Nisbet, Della Ogunleye, Anbalakan Paramasivam, Claire Searle, Adam Shaw, Eamon Sheridan, Lucy Side, Katherine Smith, Beverley Speight, William Teh, Eleanor Thorpe, Anna Whaite, Louise Wilkinson, Siobhan Wilkinson, Emma R. Woodward, Alice Youngs, Stephanie Archer, Helen Hanson

**Affiliations:** 1 St George’s University of London, London, UK; 2 Manchester Centre for Genomic Medicine, Manchester University NHS Foundation Trust, Manchester, UK; 3 Department of Medical Genetics, National Institute for Health Research Cambridge Biomedical Research Centre, University of Cambridge, Cambridge, UK; 4 Centre for Cancer Genetic Epidemiology, Department of Public Health and Primary Care, University of Cambridge, Cambridge, UK; 5 Yorkshire Regional Genetics Service, Leeds Teaching Hospitals NHS Trust, Leeds, UK; 6 Northern Ireland Regional Genetics Service, Belfast City Hospital, Belfast, UK; 7 Manchester University Hospitals; Manchester Breast Centre, Division of Cancer Sciences, Faculty of Biology, Medicine & Health, University of Manchester, Manchester, UK; 8 Maidstone and Tunbridge Wells NHS Trust, Maidstone, UK; 9 Royal Surrey NHS Foundation Trust, Guildford, UK; 10 Association of Breast Surgery, London, UK; 11 Department of Cancer Sciences, Faculty of Medicine, University of Southampton, Southampton, UK; 12 Translational Genetics Team, Division of Genetics and Epidemiology, Institute of Cancer Research, London, UK; 13 University of Southampton and University Hospital Southampton, Somers Research Building, Tremona Road, Southampton, UK; 14 Primary Care Unit, Department of Public Health and Primary Care, University of Cambridge, Cambridge, UK; 15 Department of Clinical Genetics, Royal Devon University Healthcare NHS Foundation Trust, Exeter, UK; 16 Faculty of Health and Life Sciences, University of Exeter Medical School, Exeter, UK; 17 South West Thames Centre for Genomics, St George’s University Hospitals NHS Foundation Trust, London, UK; 18 Fieldway Medical Centre, Danebury, New Addington, Croydon, UK; 19 NorthWest Thames Regional Genetics Service, Northwick Park Hospital, Harrow, London, UK; 20 Head of Screening, NHS England & Improvement (London), London, UK; 21 Northern Genetics Service, International Centre for Life, Newcastle upon Tyne, UK; 22 West of Scotland Clinical Genetics Service, Glasgow, Scotland; 23 Mermaid Centre, Royal Cornwall Hospitals NHS Trust, Truro, UK; 24 Breast Unit, East Kent Hospitals University Foundation Trust, Canterbury, UK; 25 Department of Radiology, Cambridge University Hospitals NHS Foundation Trust, Cambridge, UK; 26 Department of Radiology, University of Cambridge School of Clinical Medicine, Cambridge, UK; 27 Patient and Public Collaborators, Cambridge, UK; 28 Wessex Clinical Genetics Service, University Hospitals Southampton, Southampton, UK; 29 Southeast Scotland Genetics Service, Western General Hospital, Edinburgh, UK; 30 Clinical Genetics Department, Sheffield Children’s NHS Foundation Trust, Sheffield, UK; 31 Clinical Genetics Department, Liverpool Women’s NHS Foundation Trust, Liverpool, UK; 32 Oxford Centre for Genomic Medicine, ACE building, Nufffeld Orthopaedic Centre, Windmill Road, Headington, Oxford, UK; 33 Manchester Breast Centre, University of Manchester, Manchester, UK; 34 Nightingale Breast Unit, Wythenshawe Hospital, Manchester University Foundation Trust, Manchester, UK; 35 Public Health Commissioning and Operations, Directorate of the Chief Operating Officer, NHS England, London, UK; 36 Clinical Genetics Unit, The Royal Marsden NHS Foundation Trust, London, UK; 37 Clinical Genetics Department, University Hospitals Leicester NHS Foundation Trust, Leicester, UK; 38 Clinical Genetics Department, Great Ormond Street Hospital for Children NHS Foundation Trust, Great Ormond Street, London, UK; 39 Department of Medical Genetics, NHS Grampian, Aberdeen, UK; 40 All Wales Medical Genomics Service, Cardiff, UK; 41 Prevention & Population Research, Cancer Research UK, Cambridge, UK; 42 Clinical Genetics Department, Nottingham University Hospitals NHS Trust, Nottingham, UK; 43 Clinical Genetics Department, Guy’s and St Thomas’ NHS Foundation Trust, London, UK; 44 Genomics Unit, NHS England, London, UK; 45 Clinical Genetics Department, University Hospitals Bristol NHS Foundation Trust, Bristol, UK; 46 Clinical Genetics, Cambridge University Hospitals NHS Foundation Trust, Cambridge, UK; 47 North London Breast Screening Service, Royal Free London, London, UK; 48 Oxford Breast Imaging Service, Churchill Hospital, Oxford, UK; 49 Family History Clinic, Leeds Teaching Hospitals NHS Trust, Leeds, UK

**Keywords:** Genetic services, Cancer genetics, Risk factors

## Abstract

**Background:**

The CanRisk tool, which operationalises the Breast and Ovarian Analysis of Disease Incidence and Carrier Estimation Algorithm (BOADICEA) is used by Clinical Geneticists, Genetic Counsellors, Breast Oncologists, Surgeons and Family History Nurses for breast cancer risk assessments both nationally and internationally. There are currently no guidelines with respect to the day-to-day clinical application of CanRisk and differing inputs to the model can result in different recommendations for practice.

**Methods:**

To address this gap, the UK Cancer Genetics Group in collaboration with the Association of Breast Surgery and the CanGene-CanVar programme held a workshop on 16^th^ of May 2023, with the aim of establishing best practice guidelines.

**Results:**

Using a pre-workshop survey followed by structured discussion and in-meeting polling, we achieved consensus for UK best practice in use of CanRisk in making recommendations for breast cancer surveillance, eligibility for genetic testing and the input of available information to undertake an individualised risk assessment.

**Conclusions:**

Whilst consensus recommendations were achieved, the meeting highlighted some of the barriers limiting the use of CanRisk in clinical practice and identified areas that require further work and collaboration with relevant national bodies and policy makers to incorporate wider use of CanRisk into routine breast cancer risk assessments.

## Background

Breast cancer (BC) is the most common cancer among women worldwide [1]. BC survival has doubled in the last 50 years in the UK [2] reflecting a combination of changes in surveillance and treatment. Identifying women at increased risk of developing BC who may benefit from early and enhanced intervention (e.g. mammography, magnetic resonance imaging, chemoprevention and risk-reducing surgery) is paramount to reduce mortality [3].

The National Institute for Health and Care Excellence (NICE) guidelines for familial BC (CG164) were recently updated to reflect the need for proactive identification of women at moderate or high risk of BC within primary care [4]. Women at moderate risk of developing BC may be assessed and managed in secondary care by family history clinics in regions where these exist, whereas high-risk women should be offered a referral to a specialist genetic clinic for further assessment. According to the NICE Clinical guideline [CG164], tools such as family history questionnaires and computer packages should be made available to aid accurate collection of family history information and risk assessment [4]. However, primary care, family history clinics and Clinical Genetics services do not currently use a national, standardised tool for individualised BC risk assessment to determine eligibility for early detection strategies with breast surveillance, risk-reducing interventions such as surgery and chemoprevention and genetic testing, potentially resulting in different recommendations for clinical practice.

The most common BC risk prediction tools used in clinical practice in the UK through web-based interfaces are CanRisk (https://www.canrisk.org) and International BC Intervention Study (IBIS) or Tyrer–Cuzick models [5, 6]. Both models use cancer family history, genetic and lifestyle/hormonal risk factors to estimate a woman’s lifetime BC risk and/or the likelihood of a pathogenic variant in a BC susceptibility gene. Each model uses different risk factors and is best suited to provide risk estimates in certain patient populations[7]. Although it is outside the scope of this study to discuss BC risk prediction models in detail, Table 1 outlines some of the key differences in the two models. A detailed overview of the key characteristics of these models has been recently reviewed elsewhere [8].

**Table 1:** Differences between currently available genetic risk assessment models.

	Tyrer–Cuzick (IBIS) [6]	CanRisk [5]
**Family history of BC/OC**	First- and second-degree relatives	Beyond second-degree relatives
**Demographic factors**		
Country-specific cancer incidence	No	Yes
Birth cohort/decade of birth	No	Yes
**Lifestyle factors**		
Alcohol use	No	Yes
**Hormonal/reproductive factors**		
Oral contraceptive use	No	Yes
Age at menopause	No	Yes
**Other factors**		
History of hyperplasia/benign disease	Yes	No
History of lobular carcinoma in situ (LCIS)	Yes	No
Ductal carcinoma in situ (DCIS)	Yes	No
Breast cancer pathology/hormone status	No	Yes
**Genetic factors**		
Rare pathogenic variants in moderate and high risk breast cancer susceptibility genes	*BRCA1, BRCA2*	*BRCA1, BRCA2, PALB2, CHEK2, ATM, RAD51D, RAD51C, BARD1*
**Tools to support risk calculation**		
Software package for integration within electronic healthcare records	No	No
**Discriminatory accuracy**	Lower	Higher
CE marking	No	Since 2020

*IBIS* International Breast Cancer Intervention Study.

The CanRisk Tool is a user-friendly web-based tool that has been validated and implemented for cancer risk-stratification and prevention in primary, secondary and tertiary care [7, 9–12]. It incorporates the latest version of BOADICEA which includes established lifestyle/hormonal and clinical BC risk factors, mammographic density (MD), polygenic risk score (PRS) and pathogenic variants (PVs) in eight established BC susceptibility genes *(BRCA1, BRCA2, PALB2, CHEK2, ATM, RAD51C, RAD51D* and *BARD1)* [5, 13]. It can provide an individualised assessment to assess the likelihood of a person developing breast and/or ovarian cancer, as well as the likelihood of carrying a constitutional PV in the moderate to high-risk BC predisposition genes [11, 13, 14]. CanRisk is widely used by healthcare professionals in personalising risk assessment to facilitate shared decision-making with patients on lifestyle changes, prevention or screening options for managing BC risk. It is endorsed by multiple clinical guidelines [15].

Recent studies have highlighted the organisational barriers (e.g. available resources, integration into existing IT infrastructure, time needed to complete a risk calculation, need for training) for the wider implementation of the use of CanRisk in different clinical settings [9, 10]. To increase the consistency of the use of computer-based assessment models in clinical genetics practice across the UK, the UK Cancer Genetics Group (UKCGG) and the CanGene-CanVar programme convened a national meeting and further specific dedicated input from key stakeholders. The aims of the meeting were to:

1. Review current use of CanRisk/other computer-based models in clinical practice.
2. Discuss a consistent national approach by NHS Clinical Genetics services in the use of CanRisk to determine eligibility for breast surveillance and constitutional genetic testing.
3. To discuss a consistent national approach to use of CanRisk in determining eligibility for risk reducing mastectomy (RRM).
4. Identify where additional resource and/or pathway developments may be required.

## Methods

### Pre-meeting preparation

The organising committee (HH, MT, OT, SA, ACA, HM, GR) comprised of seven health professionals representing clinical genetics, genetic counselling and researchers, from three collaborative groups, the UK Cancer Genetics Group (UKCGG) [16], the Cancer Research UK (CRUK) funded CanGene-CanVar research programme (CGCV) [17] and the Centre for Cancer Genetic Epidemiology [18]. The focus of the meeting was the implementation of the use of CanRisk in clinical genetics practice across the UK. A preliminary survey was developed by the organising committee based on their specialist experience in this field. Invitations to register for the meeting were sent to key stakeholders and clinicians with specialist expertise and ensuring geographical representation from devolved nations. Prior to the meeting, a scoping survey (Supplementary Information) and background reading material (available at https://www.ukcgg.org/information-education/ukcgg-consensus-meetings/) were sent out to all registered delegates. This approach has been successfully used for previous UKCGG guidelines [19]. The survey questions aimed to assess current practice and to seek opinion on potential best practice pathways.

### Development of proposed statements for best practice

The themes arising from the pre-meeting survey were used to create a series of proposed statements for best practice to be addressed at the meeting. Proposed statements were developed and further reviewed by the organising committee and spanned five topics: utilisation of BC risk assessment tools, providing information to patients from CanRisk, use of CanRisk for breast surveillance recommendations and appropriate timing, use of CanRisk for genetic testing eligibility and CanRisk model inputs.

### Meeting participants

A total of 56 stakeholders registered to attend from across the UK, including patient representatives (*n* = 2), Clinical Cancer Geneticists (*n* = 22), Genetic Counsellors (*n* = 15), General Practitioners (*n* = 2), Breast Clinicians (*n* = 2), Breast Family History Leads (*n* = 2), Breast Screening Representatives (*n* = 1), Oncologists (*n* = 2), Radiologists (*n* = 3), Researchers (*n* = 3) and Programme Managers (*n* = 2). Each of the 24 UK regional genetics services were represented.

Given the relevance of CanRisk for BC family history assessments in secondary care, and difficulty in attendance at the consensus meeting, we additionally sought dedicated input into the proposed statements for best practice from five representatives of the Association of Breast Surgery (ABS).

### Format

The consensus meeting was held in Leeds on 16th May 2023 and moderated by the organising committee. The agenda for the meeting is available on the UKCGG website (https://www.ukcgg.org/information-education/ukcgg-consensus-meetings/). A series of talks from invited expert speakers were delivered on the day to provide context-specific information to delegates. The aim of these presentations, alongside pre-meeting reading materials, was to review the available evidence and the issues to be discussed at the meeting, equipping attendees with up-to-date information on which to base their votes on each proposed best practice statement.

Thereafter, a number of related polls were conducted using Slido [20], with proposed statements for best practice in different scenarios. Most polls had the option of answering on a five-point Likert scale of “strongly agree”, “agree”, “neither agree nor disagree”, “disagree” and “strongly disagree”. All attendees were allowed to vote with the exception of the three attendees from the CanRisk development team due to conflict of interest. Patient representatives and other attendees who did not feel they could answer a specific question (e.g. if a question was not applicable to their job role) were encouraged to select “unable to answer” rather than “neither agree nor disagree”. Attendees who selected “unable to answer” were excluded from the poll denominator. Each poll was closed when 80% of delegates had submitted a response. Consensus was deemed to be reached when ≥80% respondents selected “Agree/Strongly Agree” or other multiple choice poll formats in response to the statement posed; unless an argument was proposed requiring revision to the wording of the statement, after which the poll was repeated with the revised wording to generate final decision. Time was allocated for whole group discussion around each polling question for feedback, discussion and debate, which helped inform any consensus reached.

### Meeting report

After the meeting, a summary document which included the agreed best practice statements was circulated to all attendees via email for their review and comments to ensure that statements were an accurate representation of the consensus reached. It was also circulated to the representatives of the ABS for their review and feedback. The final version of the summary document is posted on the UKCGG website (https://www.ukcgg.org/information-education/ukcgg-consensus-meetings/). The consensus meeting outcomes were presented at the UKCGG 2023 Winter Meeting.

## Results and discussion

### Comments on the statements where consensus was reached

Consensus was reached on 17 of 21 proposed statements for best practice. The statements on which consensus was reached are summarised in Table 2. A consensus was not reached on four statements. Further comments on each topic follow below, using the same section headers.

**Table 2:** Statements for which consensus was reached.

**Utilisation of BC risk assessment tools**
When using a validated breast cancer risk assessment tool, there should be national consensus on which tool to use?
*(51% Strongly Agree; 45% Agree (96% consensus, n* *=* *47))*
Which breast cancer risk assessment tool do you think should be used?
*(CanRisk; 100% consensus, n* *=* *47)*
**Providing information to patients from CanRisk**
When using CanRisk, it is best practice to explain that the risk assessment could alter depending on accuracy and extent of the information input into the model and/or changes in understanding of how these factors influence risk.
*(48% Strongly Agree; 52% Agree (100% consensus, n* *=* *48))*
It is best practice to provide patients with information explaining their risk assessment in simple lay terms and in comparison to population breast cancer risk.
*70% Strongly Agree; 30% Agree (100% consensus, n* *=* *43)*
**Use of CanRisk for breast surveillance recommendations and appropriate timing**
Where appropriate infrastructure is available, use of CanRisk is the preferred method to make breast surveillance recommendations for women unaffected with breast cancer and relevant family history (where no known monogenic cause).
*(36% Strongly Agree; 55% Agree (91% consensus, n* *=* *47))*
When using CanRisk, for the purpose of a risk assessment to inform breast surveillance recommendations, it is best practice to undertake the assessment close to the age at which screening would commence i.e approaching age 40 (where no known monogenic cause).
*(14% Strongly Agree; 73% Agree (87% consensus, n* *=* *44))*
When using CanRisk, for the purpose of a risk assessment to inform breast surveillance recommendations in women with a Likely Pathogenic/Pathogenic variant in a breast cancer susceptibility gene it is best practice to undertake the assessment close to the age at which screening would commence (e.g. approaching age 25 for *BRCA1*).
*(19% Strongly Agree; 79% Agree (98% consensus, n* = *42))*
Where appropriate resource is available, it is best practice to use CanRisk to provide information on 5/10 year/lifetime breast cancer risks to women unaffected with cancer considering bilateral risk reducing mastectomy as part of broader consultation and shared decision making.
*(53% Strongly Agree; 45% Agree (98% consensus, n* = *47))*
Where appropriate resource is available, it is best practice to use CanRisk to provide information on 5/10 year/lifetime breast cancer risks to women with breast cancer considering risk reducing mastectomy as part of broader consultation and shared decision making.
*(19% Strongly Agree; 67% Agree (86% consensus, n* = *42))*
**Use of CanRisk for genetic testing eligibility**
It is best practice to use CanRisk, where the Manchester score is borderline to determine eligibility for genetic testing, where national test directory criteria are not reached based on personal history alone [33].
*(30% Strongly Agree; 68% Agree (98% consensus, n* = *44)*
When using CanRisk, it is appropriate to round up to the nearest whole number for the purpose of determining genetic test eligibility.
*(13% Strongly Agree; 70% Agree (83% consensus, n* = *46)*
**CanRisk model inputs**
Where family history is utilised, it is best practice to include at least a three-generation family tree and ensure that information on both relatives affected and unaffected with cancer is included
*(52% Strongly Agree; 48% Agree (100% consensus, n* = *40)*
When utilising CanRisk to provide an individualised risk assessment, what should represent the minimum inputs to the model for recommendations on breast surveillance (vote on all options you consider should always be used in the assessment).
*Personal History of cancer; 100% consensus, n* = *46)*
*Family History of cancer; 98% consensus, n* = *46)*
*Genetic test results (patient); 93% consensus, n* = *46)*
*Genetic test results (family member); 93% consensus, n* = *46)*
Where additional information is available for a woman beyond the agreed minimum inputs, it is best practice to utilise this information in a risk assessment.
*(33% Strongly Agree; 63% Agree (96% consensus, n* = *46))*
Where possible, all available information should be incorporated when making a risk assessment.
*(38% Strongly Agree; 58% Agree (96% consensus, n* = *45))*

### Utilisation of BC risk assessment tools

The pre-meeting survey demonstrated that in current practice many Family History clinics or Clinical Genetics services (22/33 (67%) respondents) use paper-based questionnaires to collect family history information to inform BC risk assessment. Some centres undertake assessment based on clinical criteria only (e.g age and number of affected individuals in a family). However, several multifactorial BC risk assessment models exist, each incorporating different risk factors as described elsewhere [8]. Models, such as CanRisk, that allow the combination of multiple, well-established BC risk factors (e.g., genetic/familial, demographic, lifestyle, hormonal risk factors, PRS, mammographic density etc) for risk stratification and incorporate country-specific cancer incidence data offer more accurate BC risk assessment [21].

NICE Clinical guideline [CG164] endorses rather than mandates the use of electronic risk assessment models such as BOADICEA in assessment of BC risk and genetic testing eligibility. It does not comprehensively address the situations in which it might be used e.g primary, secondary or tertiary care [22]. However, this guideline was last fully updated in 2013 and in the last 10 years there have been significant changes in the genetic testing that can be offered to families, as well as significant updates to the models. At the meeting there was strong consensus that there should be a nationally agreed model and that CanRisk is the preferred validated BC risk assessment tool to aid clinical decisions on recommendations for BC surveillance and genetic testing. CanRisk received CE marking in 2020, which is a legal requirement for medical devices being used in the European Economic Area (Table 1). At present, CanRisk is an external web-based application that requires healthcare professionals to enter data manually, which substantially adds to the time required for conducting risk assessments. However, a number of patient-facing digital family history collection tools have been developed which can reduce this burden [10, 23–26] and the CanRisk team are currently developing a patient facing tool for collecting family history and lifestyle/hormonal risk factor information (MyCanRisk).

### Providing information to patients from CanRisk

BC risk assessment using the CanRisk model is multifactorial. As with all models its accuracy will depend on the quality, validity and extent of the information available when populating the tool. CanRisk makes a number of assumptions to estimate BC risk relating to the number of known BC susceptibility genes and the information about the exact relationships among individuals within a family and is updated as and when new relevant published data becomes available [5, 13]. Consensus was reached on the importance of explaining to patients that the risk assessment could alter depending on accuracy and extent of the information input into the model and/or changes in understanding of how these factors influence risk.

Risk communication and communication of uncertainty to patients can be challenging especially in patients with low literacy [27, 28]. Explanation of risk assessment in simple lay terms and in comparison to population BC risk may support patients to make informed decisions relating to the recommended management options [29]. It is important to note that CanRisk provides risk assessment figures to age 80. Therefore, when providing patients with population BC risk figures, it is best practice to compare to population cancer risk to age 80 and not lifetime risk figures, which are higher as BC risk increases beyond this age (Table 3). The CanRisk team are in the process of adapting the CanRisk report to address these points following the consensus meeting (SA, personal communication).

**Table 3:** Summary of recommendations for the use of CanRisk in breast cancer risk assessments.

CanRisk inputs		Consensus
► At least a three-generation family tree		**√**
► Personal history of cancer		**√**
► Family history of cancer		**√**
► Genetic test results from patients		**√**
► Genetic test results from family members		**√**
► Information on both affected and unaffected relatives		**√**
► Modifiable risk factors (e.g. alcohol and weight)		**√**
► Utilise additional risk assessment information, where available		**√**
► Use only validated risk assessment information		**√**
**Providing information to patients**		
► Use simple lay terms		**√**
► Compare CanRisk outputs to population BC risk to age 80		**√**
► Explain that any changes in risk assessment information could alter CanRisk outputs		**√**
**CanRisk assessment for breast surveillance recommendations**		
► Round up CanRisk estimates to the nearest whole number		**X**
► For all women unaffected with BC with relevant family history at first appointment	• when no monogenic cause identified/known	**√**
	• with a LP/P variant in a moderate risk BC predisposition gene^a^	**√**
	• with a LP/P variant in a high risk BC predisposition gene^b^	**X**
► Repeat close to the age at which screening would commence (i.e. approaching 40 years)	• when no monogenic cause identified/known	**√**
	• with a LP/P variant in a moderate risk BC predisposition gene	**√**
► Repeat close to the age at which screening would commence (i.e. approaching 25 years)	• with a LP/P variant in a high risk BC predisposition gene	**√**
► Use the more permissive surveillance recommendation, if the 10 year risk between 40–50 differs from the lifetime risk		**X**
**CanRisk when considering Risk Reducing Mastectomy**^c^		
► To provide information on 5/10 year/lifetime or residual (from current age) BC risks	• for all women unaffected with BC	**√**
	• for all women affected with BC	**√**
► To provide information on contralateral BC risk	• for all affected women with unilateral BC	**√**
► Clinical Genetics input should be mandatory	Discussion in joint RRM/Clinical Genetics MDT, where available	**√**
**CanRisk to determine genetic test eligibility**		
► Round up CanRisk estimates to the nearest whole number		**√**
► When the Manchester score is borderline (e.g. 13–14) (R208 panel; criteria 1 f)		**√**

^a^*CHEK2*, *ATM*, *RAD51C*, *RAD51D* and *BARD1*.

^b^*BRCA1*, *BRCA2* and *PALB2* (entry to Very High Risk Screening Programme age 30 is not dependant on individualised risk assessment).

^c^in conjunction with appropriate counselling of the potential limitations of the tool and shared decision making.

### Use of CanRisk for breast surveillance recommendations and appropriate timing

CanRisk is a comprehensive risk prediction model offering personalised risk assessment in comparison to more traditionally utilised clinical algorithms based solely on family history. A greater proportion of delegates considered CanRisk the preferred method to help direct unaffected women with a relevant family history, where genetic testing is not indicated or where genetic testing does not identify a causative variant, to the most appropriate breast surveillance in conjunction with appropriate counselling and discussion of the risk assessment. During discussions it was highlighted that risk estimates based on multifactorial risk models may be associated with wide confidence intervals based on the uncertainty related to input parameters. Therefore, responsible clinicians should explain to patients the limitations of risk prediction tools and use these as an adjunct to discussions with individual patients with respect to discussion of BC risk, potential lifestyle changes and/or surveillance options.

According to the current NICE Clinical guideline [CG164], changes to a woman’s family history should prompt a repeat BC risk assessment to inform breast surveillance recommendations [22]. In addition to family history, changes in lifestyle or hormonal factors included in risk prediction tools could also potentially alter an individual’s risk reassessment. Delegates discussed whether women should be advised to seek a referral for an up-to-date assessment close to the age at which breast surveillance would commence in order to provide the most up to date assessment and advice. Although local services need to be taken into consideration, a greater proportion of delegates considered a risk assessment using CanRisk close to the age at which surveillance should commence was most appropriate (e.g. approaching age 40 in the absence of a pathogenic variant or approaching age 25 for women with a Likely Pathogenic/Pathogenic (LP/P) variant in a high-risk BC predisposition gene).

Consensus was not reached on the utility of CanRisk as a method to make breast surveillance recommendations in unaffected women with a LP/P variant in a BC predisposition gene. In England, for high-risk genes (e.g., *BRCA1, BRCA2* and *PALB2*) entry into the Very High Risk Breast Screening (VHRS) Programme at age 30 is not dependant on an individualised assessment, given the high lifetime risks associated with these genes. Therefore, a number of delegates felt a CanRisk assessment would take up time and resource and was not required for these individuals unless personalised risk assessment would aid discussions on 5-year, 10-year and lifetime risk and/or risk reducing options (Table 3). Although not specifically discussed at the meeting, a CanRisk assessment is currently required for *BRCA1*, *BRCA2* and *PALB2* carriers between ages 25–30 to qualify for entry into the VHRS programme based on defined 10-year risk estimates [30].

Some delegates were supportive of a CanRisk assessment to help direct women with LP/P variants in moderate risk genes to the most appropriate breast surveillance, given that family history and other factors can strongly influence lifetime BC risk in moderate risk gene carriers. However, other delegates felt that an assessment may not result in a change in practice and may not therefore represent best use of clinical time. There was agreement that automation of assessment may make this more feasible in the near future. Consensus was reached that a personalized risk assessment is warranted for women unaffected with BC who have a LP/P variant in a moderate risk BC predisposition gene (e.g., *CHEK2, ATM, RAD51C, RAD51D* and *BARD1*) similar to unaffected women with relevant family history and no known monogenic cause (Table 3).

CanRisk automatically provides both 10-year BC risk between 40–50 and lifetime BC risks in the output and in some situations this can result in differing surveillance recommendations when applying NICE guidelines. There was discussion in the meeting about the advantages/disadvantages of choosing the more permissive surveillance recommendation to ensure that centres are being consistent with their recommendations. Although consensus was not reached on this best practice statement (Table 3), it was felt by some delegates that the risk for the next 10 years would be the most clinically relevant compared to the lifetime risk and is the risk utilised for VHRS recommendations. Therefore, it was suggested that this best practice statement might be best addressed in future updates to NICE Clinical guideline [CG164].

### CanRisk when considering bilateral risk reducing mastectomy

CanRisk provides information on 5 year and 10 year BC risk from current age, on lifetime BC risk from 20 to 80 years, as well as on residual lifetime BC risk from current age of patient (e.g. if a woman is 52 years, then it will give the BC risk until age 80). There was consensus that risk information provided by CanRisk on 5/10 year/lifetime and residual BC risks should be used as an adjunct to a broader consultation when women, affected or unaffected with cancer, are considering RRM and in surgical decision making. Such cases should ideally be discussed in a joint RRM/Clinical Genetics multidisciplinary meeting (MDT). Where joint MDT is not possible, opinion from Clinical Genetics should be mandatory in the risk assessment process (Table 3).

CanRisk can also provide information on contralateral BC risk for a woman who has had a unilateral BC, which is an important discussion point in surgical decision making. However, estimation of contralateral risk is a challenge because there are limitations of the CanRisk tool in predicting contralateral BC risk, as it does not include all the relevant competing/treatment factors. CanRisk does not take account of risk reduction from adjuvant therapies for contralateral risk (e.g. endocrine therapy or anti-HER2 chemotherapy). In addition, CanRisk does not currently take account of prognosis of primary tumour. It was also acknowledged that there is limited data on contralateral BC risk outside *BRCA1* and *BRCA2*. Recent studies have confirmed that contralateral risk is likely increased for *CHEK2* carriers but may not be increased for *ATM* carriers [31, 32]. It is clear that further studies are needed, and even when clearer risks have been established, these will then need to be incorporated into the relevant risk models. Specifically for women affected with unilateral BC, it was felt that CanRisk assessment should not be used in isolation and full evaluation of previous treatment and competing risks should be undertaken. These cases should be discussed in a breast MDT and where possible joint MDT with Clinical Genetics (Table 3).

### Use of CanRisk for genetic testing eligibility

According to the National Genomic Test Directory, a living affected individual (proband) with breast or high-grade ovarian cancer who meets certain testing criteria based on personal history and/or family history should be eligible for the Inherited BC and ovarian cancer (R208) gene panel testing, which currently includes *BRCA1, BRCA2, PALB2, ATM, CHEK2, RAD51C* and *RAD51D* [33]. In certain cases, healthcare professionals may need to calculate a Manchester Score (MS) to determine a patient’s genetic testing eligibility (criteria 1 f R208). This is a simple scoring system that includes information on family history and tumour pathology to calculate the likelihood of identifying patients with a *BRCA1* or *BRCA2* pathogenic variant [34–36]. Patients with an MS of ≥ 15 are eligible for R208 testing. As MS is only based on family history and on likelihood of *BRCA1/2* only, some individuals may be eligible for testing if CanRisk is alternatively used to assess eligibility for testing. Conversely, as CanRisk also utilises information on number of unaffected relatives in a family and other risk factors for BC, some patients may reach eligibility on MS, but not CanRisk. Whilst it would be most comprehensive to calculate both MS and CanRisk for all patients to assess eligibility, this is not currently possible due to the resources required. At present, MS is most routinely used in clinical practice given it is a quick and simple score to calculate. Whilst it was recognised that routine CanRisk assessment is not currently possible for all patients, consensus was reached that it should be considered for those individuals with a borderline MS (i.e. 13/14), where eligibility for testing may be reached on utilisation of CanRisk.

CanRisk outputs for BC surveillance recommendations or for genetic testing eligibility are detailed to one decimal place. However, it is likely there are confidence intervals for these risk assessments, which are not detailed on the output report, and thus, recommendations are based on a single figure. There was much discussion about how to address this in practice, with some delegates strongly supportive of a more permissive approach and rounding up to the nearest whole number, whereas others felt that providing accurate information was inputted into the model, the figure should be utilised as it stands. There were also concerns raised over the illusion of certainty that one decimal place may convey to clinicians and patients. Although consensus was reached to support rounding up CanRisk estimates to determine genetic test eligibility, consensus was not reached for rounding up CanRisk estimate in the situation of breast surveillance recommendations (Table 3).

### Altering the default test sensitivity settings in CanRisk

There was also discussion in the meeting about the sensitivity of genetic testing and how to use this information in a risk assessment. Sensitivity relates to how well the genetic test undertaken detects a genetic variant in a specific gene. In a CanRisk assessment, when estimating the residual likelihood of a pathogenic variant in a family and/or BC risk, the sensitivity of testing matters only in cases where no pathogenic variant is identified during a “mutation search” (i.e a diagnostic test that is analysing a whole gene, rather than a targeted test for a familial variant). This may indicate the possibility that a pathogenic variant might have been missed by the genetic test. CanRisk uses default genetic test sensitivities, which are based on the genetic technologies used at the time the model was developed [13]. The genetic test sensitivity depends on when the genetic test was undertaken, and the type of technology used to perform the test and therefore may differ from the default setting in CanRisk. For example, at present the default sensitivity setting for *BRCA1* is 89%. However, in current practice sensitivity of testing is typically at least 95% using NGS-based technology [37]. A polling question regarding altering the default test sensitivity settings in CanRisk according to the sensitivity of the test performed showed that a greater proportion of delegates considered this appropriate where genetic testing of one or more BC predisposition genes has been undertaken in a family member. However, in meeting discussions revealed that non-geneticists, including breast surgeons and family history clinics, do not have sufficient expertise to alter the default sensitivity settings in CanRisk. Therefore, it was suggested that the default settings in CanRisk should be set to reflect current sensitivity of testing. It was agreed that the UKCGG will liaise with the Genomic Laboratory Hubs and the Association for Clinical Genomic Science (ACGS) to propose specific genetic test sensitivity figures based on current technologies and update the default settings in CanRisk, rather than require users to alter the sensitivity settings (Table 4).

**Table 4:** Identified areas for further work.

Identify the required resources to implement CanRisk across the UK Clinical Genetics services.	UKCGG
Identify the required resources to implement CanRisk across Family History clinics and Breast services.	UKCGG, ABS
Provide healthcare professionals with the additional training required to use the CanRisk tool.	ABS, UKCGG, CanRisk team
Update the default sensitivity settings in CanRisk based on current technologies.	UKCGG/ACGS/CanRisk Team
Seek Radiology input regarding rounding up CanRisk estimates to the nearest whole number for the purposes of making breast surveillance recommendations.	UKCGG, ABS, The Royal College of Radiologists
Seek Radiology input for the implementation of a national pathway to incorporate mammographic breast density in routine BC risk assessment.	UKCGG, ABS, The Royal College of Radiologists, National Screening Committee
Review and update of the Risk Reducing surgery guidance.	ABS, UKCGG
Liaise with NICE to consider an update to CG164 regarding the use of CanRisk into clinical practice.	UKCGG, ABS

### CanRisk model inputs

CanRisk provides an individualised risk assessment by incorporating personal and family history of cancer, as well as predictive and diagnostic genetic test results. In addition, hormonal and lifestyle factors can modify BC risk, which can be helpful when counselling patients and in shared decision making. Other factors such as mammographic density and PRS also modify risk and if included in an assessment could alter recommendations for surveillance [12, 38–40]. However, both PRS and mammographic density are not currently available in routine clinical practice. The in-meeting poll allowed delegates to select the inputs that they consider should always be used in BC risk assessments as a minimum for recommendations on breast surveillance. Consensus was reached to consider, as minimum inputs: (i) the personal and family history of cancer and (ii) a patient’s or a family member’s genetic test result. It was agreed at the meeting that a three-generation family tree should be used in all CanRisk assessments including information on both affected and unaffected relatives. Although not specifically discussed at the meeting, given the potential for misreporting of family history of cancer, particularly for ovarian cancer [41, 42], routine clinical practice should include verification of personal and family history of cancer, particularly for abdominal malignancies and in situations where risk reducing surgery is considered [22, 43]. Confirmation is typically sought for diagnoses of OC, bilateral BC or very young onset BC where decisions on genetic testing may be impacted. Where risk reducing mastectomy is being considered for a family history of BC in the absence of a PV, confirmation of BC diagnoses in the family should be considered mandatory [22]. Whilst death certificates can be requested by patients, confirmation via National Cancer Registry data through Genetics services may be required.

Whilst there was consensus to include all relevant information where possible when making a risk assessment, the meeting discussions and subsequent comments demonstrated that the statement was too broad for consideration. Therefore, it was agreed that only appropriately confirmed input information should be incorporated in a CanRisk assessment (e.g, use of PRS when approved methodology has been followed or as part of a research study).

### Alcohol and weight

The incorporation of modifiable risk factors such as alcohol and weight should be included in the CanRisk model, where available. They should also be included in discussions when counselling patients regarding lifestyle changes for cancer prevention. However, it was noted that personal assessments of alcohol intake are very unreliable, and the accuracy of the reported information should be considered. Overall, it was felt that the validity and confidence in the input information provided should be clinician guided.

### Mammographic density and PRS

Mammographic breast density and PRS are not currently routinely offered in the National Health Service (NHS) and there were discussions about utilising these risk factors, if they had been obtained through a different setting e.g private practice/overseas practice or research studies. There were both concerns about widening inequity if these factors are used in a risk assessment for patients where they are available, balanced with opinions that all validated available information should be utilised to provide a more personalised risk assessment.

During the meeting it was discussed that mammographic breast density is considered an important input to determine breast surveillance and should be used in CanRisk assessment of BC risks, where available. However, only 10% (5/48) of the attendees voted that mammographic density should always be used in the CanRisk assessment. The lack of consensus in this area relates to the fact that information on mammographic density is not widely available. Furthermore, women have not generally had a mammogram when they come forward for family history or genetics assessment and there is no pathway for reassessment after a mammogram. Importantly, manual mammographic density assessment is subjective and no automated approach for measuring breast density is currently in routine use. Therefore, if mammographic density were to be routinely utilised in BC risk assessment, implementation of a new national pathway would be required (Table 4).

Similarly, there was reluctance to use PRS in a risk assessment at the current time. Genetic testing for PRS is not currently routinely available in the NHS. Two research studies led by the University of Cambridge are assessing the acceptability and feasibility of PRS in unaffected women with a family history of BC referred to Clinical Genetics services (CanRisk-ClinGen) and in unaffected women identified to have a familial pathogenic variant in a BC predisposition gene (Precision-HBOC study [44]). It was widely agreed that prior to any recommendation on the introduction of PRS in routine clinical practice, there should be accredited, or kite marked validated assays or NEQAS approved NHS laboratory assays and that non validated assays should not be included in a CanRisk assessment outside clinical trial context at this stage.

## Strengths, limitations and future directions

This is the first UK meeting to address the challenges in using CanRisk in clinical practice. The expertise of the group members enriched the discussions allowing the group to reach consensus on best practice guidelines relating to the indications of the use of CanRisk for BC surveillance recommendations and genetic testing eligibility and the input of available information into CanRisk for an individualised risk assessment (Table 3).

Whilst there is support to use CanRisk nationally, many centres are limited by lack of resources. The pre-meeting survey demonstrated that only 36.4% (12/33 respondents) of Clinical Genetics services have the appropriate resources to implement the wider use of CanRisk and highlighted some of the barriers limiting the use of CanRisk, including workforce capacity, staff education and access to funding, as well as concerns over consistency of use of mammographic density and PRS data across the regions. Moreover, the CanRisk tool is not integrated into existing IT infrastructure and many Family History clinics or Clinical Genetics services (22/33 (67%)) use paper questionnaires when collecting data for BC risk assessments. Therefore, changes to infrastructure and pathways are required to implement CanRisk more widely. In particular, the development of patient facing data collection tools (e.g. MyCanRisk) that integrate with CanRisk and allow patients to enter their risk factor information and family history online may reduce the burden on clinical services, providing that risk assessment outputs are used to aid discussions and do not replace clinical judgement and decision making.

The consensus meeting identified areas that require further work and collaboration with relevant national bodies and policy makers (Table 4). The NICE Clinical guideline [CG164] is due for review and there has been significant evolution of our understanding of BC predisposition since the last update [22]. Therefore, we would encourage any updates to make more specific recommendations on the use of digital assessment tools considering both resource implication and pathway development.

### Supplementary information

Supplementary information

## Data Availability

The datasets generated and/or analysed during the current study are available from the corresponding author on reasonable request.
