# Supplementary material for: Joint ABS-UKCGG-CanGene-CanVar consensus regarding the use of CanRisk in clinical practice
Source: Br J Cancer. 2024 Jun 4;130(12):2027–36. doi: 10.1038/s41416-024-02733-4 (PMC11183136; doi:10.1038/s41416-024-02733-4)
Supplement: Supplementary file 1 — Supplementary information [file 41416_2024_2733_MOESM1_ESM.docx]

**Supplementary information: Questions of the scoping survey sent out to all registered meeting delegates to assess current practice and to seek opinion on potential best practice pathways relating to the use of CanRisk.**

**UK CGG CanRisk Survey**

**Page 1: Background information**

**Q1 What is your job title?**

Free text box

**Q2 Where is your place of work?**

Clinical Genetics

Family History Clinic

Breast Clinic

Other (please specify)

**Q3 What is the name of the hospital/centre where you work?**

Free text box

**Page 2: Current practice**

**Q4 When do you most often collect family history information for patients referred into your service regarding a family history of breast +/- ovarian cancer, without a known familial variant in a cancer predisposition gene?**

Pre-appointment

During appointment

Following appointment

Not applicable to my job role

Other (please specify in the free text)

Free text box

**Q5 When do you most often collect family history information for patients referred into your service regarding a family history of breast +/- ovarian cancer, with a known familial variant in a cancer predisposition gene (i.e for predictive testing)?**

Pre-appointment

During appointment

Following appointment

Not applicable to my job role

Other (please specify in the free text)

Free text box

**Q6 In what format do you currently collect family history information?**

Paper questionnaire

Online questionnaire

Direct input into CanRisk/ or other online assessment mode

Not applicable to my job role

Other (please specify in the free text)

Free text box

**Q7 What information do you currently ask for as part of the family history questionnaire? Please tick all that apply.**

Personal history of cancer

Family history of cancer

Age of menarche

Age of menopause

Use of OCP

Use of HRT

Height

Weight

Alcohol

Smoking history

Mammographic density

Tubal ligation

Endometriosis

Results of genetic testing

Polygenic risk score

Other (please state)

**Q8 How do you currently determine eligibility for breast screening in a woman unaffected with breast cancer with no known pathogenic/likely pathogenic variants in breast cancer predisposition genes?**

CanRisk

Tyrer Cuzick

Clinical criteria (Please state which one in free text)

Other (please state in the free text)

Free text

**Q9 How do you currently determine breast cancer risk in an unaffected woman with a BRCA1, BRCA2, PALB2, RAD51C, RAD51D, ATM or CHEK2 pathogenic variant in order to guide future surveillance and/or risk reducing surgery?**

*BRCA1*

*BRCA2*

*PALB2*

*CHEK2*

*ATM*

*RAD51C*

*RAD51D*

CanRisk

Tyrer Cuzick

Clinical criteria (Please state which one in free text)

Other (please state in the free text)

Free text

**Q10 Following on from Q9, does the age of the patient impact your decision on how to assess risk?**

Yes

No

Other (please specify in the free text)

Free text

**Q11 How do you currently determine contralateral breast cancer risk to guide future surveillance and/or risk reducing surgery in a woman who has been affected with breast cancer and has *a BRCA1, BRCA2, PALB2, RAD51C, RAD51D, ATM* or *CHEK2* pathogenic variant?**

*BRCA1*

*BRCA2*

*PALB2*

*CHEK2*

*ATM*

*RAD51C*

*RAD51D*

CanRisk

Tyrer Cuzick

Clinical criteria (Please state which one in free text)

Other (please state in the free text)

Free text

**Q12 Following on from Q11, does the age of the patient impact your decision on how to assess risk?**

Yes

No

Other (please specify in the free text)

Free text

**Q13 Using CanRisk estimates to one decimal place is appropriate to determine breast screening.**

Strongly agree

Agree

Neither agree nor disagree

Disagree

Strongly disagree

Any other comment

**Q14 Rounding up or down of estimates generated from CanRisk should be allowed to determine breast screening?**

Strongly agree

Agree

Neither agree nor disagree

Disagree

Strongly disagree

Any other comment

**Q15 Where genetic testing of one or more breast cancer predisposition genes has been undertaken in a family member, do you alter the default test sensitivity settings to determine breast cancer risk in family members?**

Yes. If yes, what setting they are altered to? Please state in free text below.

No

Other (please specify in the free text)

Free text

**Q16 When determining eligibility the breast cancer predisposition panel R208 , how do you assess eligibility for criteria 1f, where eligibility is based on personal and family history: combined pathology-adjusted Manchester score ≥15 or single gene pathology adjusted score of ≥10 or BOADICEA/CanRisk score ≥10%?**

CanRisk

Manchester score

Clinical criteria (please state which one in free text below)

Combination of above

Other (please specify in the free text)

Free text

**Q17 Using CanRisk estimates to one decimal place is appropriate to determine eligibility for genetic  testing.**

Strongly agree

Agree

Neither agree nor disagree

Disagree

Strongly disagree

Any other comment

**Q18 Rounding up or down of estimates generated from CanRisk should be allowed to determine eligibility for genetic testing?**

Strongly agree

Agree

Neither agree nor disagree

Disagree

Strongly disagree

Any other comment

**Page 3: Resource questions**

**Q19 Does your service currently have the infrastructure/resource to implement CanRisk for all breast cancer family history assessments performed through clinical genetics?**

Yes

No

Uncertain

Other (please specify in the free text)

Free text

**Q20 Do family history/secondary care services associated with your Genetics service currently have the infrastructure/resource to implement CanRisk for all breast cancer family history assessments?**

Yes

No

Uncertain

Other (please specify in the free text)

Free text

**Q21 What are the factors limiting your service, if you do not currently use CanRisk?**

Infrastructure/resources

Lack of confidence in model

Concern about consistency of use across the region

Please use free text to expand below

**Page 4: Future practice**

**Q22 We should use CanRisk for all breast cancer risk assessments in unaffected women (assuming appropriate resource was available) to determine future breast cancer risk.**

Strongly agree

Agree

Neither agree nor disagree

Disagree

Strongly disagree

Any other comment

**Q23 We should use CanRisk for all assessments in women affected with breast cancer (assuming appropriate resource was available) to determine contralateral breast cancer risk.**

Strongly agree

Agree

Neither agree nor disagree

Disagree

Strongly disagree

Any other comment

**Q24 We should use CanRisk for all genetic testing assessments in women affected with breast cancer (assuming appropriate resource was available) to determine eligibility for genetic testing if criteria are not reached on personal diagnosis (i.e criteria 1f for R208).**

Strongly agree

Agree

Neither agree nor disagree

Disagree

Strongly disagree

Any other comment

**Q25 Where possible, CanRisk should be used for all breast cancer risk assessments in *BRCA1, BRCA2, PALB2, ATM, CHEK2, RAD51C, RAD51D* gene carriers (assuming appropriate resource was available) to determine future breast cancer risk and appropriate breast screening/risk reducing surgery options.**

Strongly agree

Agree

Neither agree nor disagree

Disagree

Strongly disagree

Any other comment

**Q26 Where possible, CanRisk should be used for all ovarian cancer risk assessments in *BRCA1, BRCA2, PALB2, RAD51C, RAD51D* gene carriers (assuming appropriate resource was available) to determine future ovarian cancer risk and to inform risk reducing surgery options.**

Strongly agree

Agree

Neither agree nor disagree

Disagree

Strongly disagree

Any other comment

**Q27 There should be a consistent national approach regarding CanRisk inputs?**

Strongly agree

Agree

Neither agree nor disagree

Disagree

Strongly disagree

Any other comment

**Q28** **Which of the following inputs should we use as a minimum for determining breast screening category in CanRisk (tick as many as you think are required for minimum assessment)?**

Personal history of cancer

Family history of cancer

Age of menarche

Age of menopause

Use of OCP

Use of HRT

Height

Weight

Alcohol

Mammographic density

Results of genetic testing

Polygenic risk score

Any other comment

**Q29 Where PRS is available this should be used in CanRisk assessment of breast cancer risks?**

Strongly agree

Agree

Neither agree nor disagree

Disagree

Strongly disagree

Any other comment

**Q30 Where mammographic density is available, this should be used in CanRisk assessment of breast cancer risks?**

Strongly agree

Agree

Neither agree nor disagree

Disagree

Strongly disagree

Any other comment

**Q31 All the information available for a woman at that point in time, should be used in a Canrisk assessment recognising that this could create in consistency in risk assessment for women where that information is not available.**

Strongly agree

Agree

Neither agree nor disagree

Disagree

Strongly disagree

Any other comment

**Q32 How often should a Canrisk be repeated for a woman?**

It should be undertaken regularly, e.g. every 5 years

It should be undertaken if a change in personal or family history

It should be undertaken prior to the point of entry of a surveillance programme e.g at 40 years for women who have moderate risk surveillance

It should be undertaken prior to the point of exit of a surveillance programme e.g at 50 years for women who have moderate risk surveillance

Other (please specify)

**Q33 Thank you for completing the survey. Please use the box below if you have any other comments.**

Free box for comments
